# A Comparative Study of Premixed Versus Succedent Administration of Dexmedetomidine and Bupivacaine in the Subarachnoid Block for Infraumbilical Surgeries

**DOI:** 10.7759/cureus.93536

**Published:** 2025-09-30

**Authors:** Lily S Bodra, Dipali Singh, Bharati Bharati, Shio Priye, Saurabh Toppo, Sourabh Kumar

**Affiliations:** 1 Department of Anaesthesiology, MGM Medical College and Hospital, Jamshedpur, IND; 2 Department of Anaesthesiology, Rajendra Institute of Medical Sciences, Ranchi, IND; 3 Department of Anaesthesiology, Bhagwan Mahavir Manipal Hospital, Ranchi, IND

**Keywords:** bupivacaine, dexmedetomidine, premixed, subarachnoid block, succedent

## Abstract

Background: The suggested method for infraumbilical surgeries is spinal anaesthesia. Postoperative pain management is challenging, as spinal anaesthesia utilising solely local anaesthetics provides only a short analgesic effect. Therefore, early pain relief is necessary in the postoperative period.

Aims and objectives: In this study, dexmedetomidine and bupivacaine were administered either premixed in a single syringe or sequentially in separate syringes for subarachnoid block in infraumbilical surgeries across different study groups. We compared the sensory and motor block characteristics, intra-operative hemodynamics, postoperative pain relief, and any side-effects in patients undergoing lower abdominal surgeries. The effect of administering dexmedetomidine before bupivacaine heavy and vice versa on these parameters was also assessed.

Methodology: The study involved 120 patients, as classified by the "American Society of Anaesthesiologists", physical status I and II, all scheduled for lower abdominal procedures (gynaecological, urological, orthopaedic, and other lower abdominal procedures) under spinal anaesthesia. Three groups of patients were randomly selected: Group A received two intrathecal injections. The first injection consisted of 5 mcg of dexmedetomidine diluted in preservative-free normal saline (0.5 mL), followed by 12.5 mg of 0.5% heavy bupivacaine (2.5 mL), both in separate 3 mL syringes. In Group B, participants received intrathecal injections of premixed solution divided into two separate 3 mL syringes, one with 2.5 mL and the other with 0.5 mL of premixed solution. A premixed solution (3 mL) was made with 5 mcg of dexmedetomidine diluted in 0.5 mL of preservative-free normal saline and 12.5 mg of 0.5% heavy bupivacaine (2.5 mL). Group C participants were given two consecutive intrathecal injections, first 12.5 mg of 0.5% heavy bupivacaine (2.5 mL) using a 3 mL syringe and 5 mcg of dexmedetomidine diluted in preservative-free normal saline (0.5 mL) in a second 3 mL syringe. Statistical analysis has been performed utilizing Statistical Product and Service Solutions (SPSS, version 20; IBM SPSS Statistics for Windows, Armonk, NY).

Results: The earliest average onset time for sensory block has been observed in Group A, which was monitored by Groups B and C. Group C had the lowest average onset time for motor block, followed by Groups A and B. Groups A and C experienced sensory and motor blockage for more extended periods. Compared to Groups A and C, Group B patients have a greater incidence of bradycardia as well as hypotension.

Conclusions: The research illustrates that, in contrast to spinal anaesthesia with a premixed dosage of hyperbaric bupivacaine and dexmedetomidine, sequential intrathecal injection produces a longer sensory and motor block with a quicker onset.

## Introduction

The most important aspect of surgical patients' treatment is to manage their postoperative pain. An increase in death and morbidity may result from inadequate pain management. Intraoperative, preoperative, and postoperative treatment of patients falls under the responsibility of the modern anaesthesiologist. There is an increasing interest in replacing general anaesthesia with regional anaesthesia methods for several standard surgical procedures. Regional anaesthesia provides numerous advantages over general anaesthesia, including less intraoperative haemorrhage, enhanced muscle relaxation, and the reduction of pain during and after surgery [1]. Additionally, the adverse effects and analgesic profile of regional anaesthetic operations are superior to the effects of systemic opioid medications. Because of its reliability, affordability, and ease of application, spinal anaesthesia is the most widely used method [1]. Research indicates that spinal anaesthesia, in place of general anaesthesia, leads to a reduced hospital stay, increased compliance, and faster recovery of many physiological functions following abdominal and gynaecological procedures [2].

The earliest description of spinal anaesthesia was made more than a century ago. Neuraxial drug delivery has consequently quickly advanced and currently includes a variety of drugs that offer both analgesia and anaesthesia. With an eight times alpha 2/alpha 1 selectivity ratio higher than clonidine's, dexmedetomidine is a recently developed alpha-2 adrenergic receptor agonist that is incredibly selective [3]. Although there is not much research on the subject, several authors have discussed using intrathecal dexmedetomidine, as well as spinal local anaesthetics. By binding and preventing the release of presynaptic C-fibre neurotransmitters, as well as hyperpolarising postsynaptic dorsal horn neurones, intracerebral alpha 2-adrenoceptor agonists provide analgesia. Alpha-2 adrenoceptor agonists prolong motor block by attaching to dorsal horn motor neurons; this anti-nociceptive activity may also prolong sensory block. Additionally, the Food and Drug Administration (FDA) authorised the use of dexmedetomidine as a sedative and short-term analgesic for patients in intensive care units (ICUs) undergoing ventilation in 1999. Its use has been associated with a reduction in blood pressure (BP) and heart rate (HR). Neurological abnormalities were not observed in any of the numerous studies conducted on animals administered intrathecal dexmedetomidine at dosages ranging from 2.5 to 100 mcg [4,5]. This drug has been researched as a neuraxial adjuvant because of its excellent intraoperative and postoperative analgesia, stable haemodynamic conditions, and few invasive side effects. In patients undergoing infra-umbilical surgeries, we aimed to use dexmedetomidine as a supplement to the intrathecal block caused by bupivacaine because of its numerous advantages. Gupta et al. [6] examined 60 individuals undergoing surgery on the lower abdomen. The hyperbaric solution each patient received either 12.5 mg of bupivacaine plus 25 μg intrathecal fentanyl or 5 μg dexmedetomidine at random. Sensory regression to S1 required an average of 476±23 minutes in Group D and 187±12 minutes in Group F (p < 0.001). It took 421±21 minutes for Group D to reach modified Bromage 0, but Group F required 149±18 minutes (p<0.001). Using bupivacaine and adjuvants along with fentanyl, as well as nalbuphine, Birajdar et al. [7] studied the onset, duration, and side effects of epidural analgesia during lower limb procedures. They concluded that epidural fentanyl acts faster and lasts longer than nalbuphine for postoperative epidural analgesia.

Local anaesthetics (LA) intrathecal distribution has been discovered to be affected by baricity, drug pH, temperature, and the posture of the patient after injection. During invasive procedures, the impact of levobupivacaine alone and in combination with fentanyl has been studied by Attri et al. [8]. They concluded that fentanyl and levobupivacaine produce surgical analgesia with consistent haemodynamics as well as few side effects and sensory and motor block that begins early and lasts for an extended period of time. Chaudhary et al. [9] have investigated the effectiveness of premixed versus sequential dexmedetomidine distribution as a supplement to intrathecal hyperbaric bupivacaine. They came to the conclusion that the haemodynamic parameters, postoperative analgesic demand, side effect profile, and block characteristics of the two groups differed.

LA and adjuvants have been frequently combined in one syringe before the drugs are injected intrathecally. When some drugs are used together, their densities change, which affects how the molecules distribute throughout the cerebrospinal fluid (CSF). LA dispersion is known to be determined by density; however, nothing is known about how adjuvant solution density impacts LA mobility in CSF. Therefore, we reasoned that, if LA and adjuvants were administered independently, density strength would alter and, therefore, their activities may be decreased [10]. Subarachnoid block (SAB) given in patients undergoing lower abdominal surgeries was assessed for intra-operative hemodynamics, block properties, and postoperative pain control following the sequential use of hyperbaric mixtures of bupivacaine (HB), as well as dexmedetomidine in one or two syringes. The research aims to assess the efficacy of delivery of hyperbaric bupivacaine intrathecally as well as dexmedetomidine for lower abdominal procedures, both separately and in combination. Time to rescue analgesia, adverse effects, and haemodynamic stability were also examined.

## Materials and methods

Study design

Prospective, randomised, double-blind examination was conducted over one and a half years in a tertiary care hospital in Jharkhand, following institutional research and ethical committee permission. Research has been accepted by the Institutional Ethics Committee (IEC) using CTRI-CTRI/2021/11/037999 and IEC memo no 105, dated 10/04/2021.

Data collection

Our inclusion criteria include American Society of Anaesthesiologists (ASA) grade I and II patients, age between 18 and 60 years, weight between 40 and 70 kg, and providing written informed consent, along with elective infraumbilical procedures lasting no more than two hours. Patients with ASA>II, allergies to local anaesthetics, patient's refusal, and any condition that prevents spinal anaesthesia were the criteria for exclusion.

In this study, a total of 120 patients, 40 in each group, scheduled for infraumbilical procedures under spinal anaesthesia, were included. On the day before the procedure, there was a pre-anaesthetic check-up that included a complete history, a systemic examination, and a thorough physical examination. All routine tests were performed, including a posterior-anterior chest radiograph, random serum creatinine, blood sugar, ECG, serum urea, a complete blood count, and a routine urine test. Every patient was randomly assigned to one of three groups of 40. Written informed consent has been provided by patients. Following premedication, every patient was moved to the operating room, where baseline measurements were made, including BP, oxygen saturation (SPO_2_), respiratory rate (RR), HR, and ECG.

Group A received intrathecal injections of 5 mcg of dexmedetomidine diluted with preservative-free normal saline (0.5 mL) in a 3 mL syringe, followed by 12.5 mg of 0.5% heavy bupivacaine (2.5 mL) in a second 3 mL syringe. Group B received an injection of a premixed drug that contained 2.5 mL of 12.5 mg of 0.5% heavy bupivacaine and 5 mcg of dexmedetomidine diluted with 0.5 mL of normal saline without any preservatives. After that, the mixture was split into two different 3 mL syringes, one containing 2.5 mL and the other containing 0.5 mL. Group C was given intrathecal injections of 12.5 mg of 0.5% heavy bupivacaine (2.5 mL) in a 3 mL syringe, followed by 5 mcg dexmedetomidine diluted with preservative-free normal saline (0.5 mL) in a second 3 mL syringe. The accompanying anaesthesiologist, who was not involved in this study, was then given the medication in a coded form. A 25 G Quincke needle was used to give spinal anaesthesia to all patients in a seated posture at L3-4 utilizing a midline approach, and the drug was given based on group-allocated patients. The subarachnoid block was given. Patients were then placed in the supine position after receiving a spinal injection. All patients were given oxygen with face masks. The duration required for the highest sensory level to be reached, the regression of motor block, the beginning of sensory or motor block, and the two dermatome level regression from maximum sensory block were noted.

A blunted needle pinprick was used to assess the sensory block until the highest level was reached. The onset of sensory block was concluded by the lack of sensation at the T10 dermatome after injection. From the maximum block height to two dermatomal level regression, it calculates the length of sensory block regression. Motor block was assessed using the modified Bromage scale [11] as follows: While performing full straight leg lifts, 0 reported no block; one person reported being unable to lift the straight leg while flexing the knee; two people said they could flex the ankle but not the knee; and three reported having whole motor movement and total block. The duration required for a motor block to regress from a 3 to 0 scale was determined using the modified Bromage scale. Values for intraoperative hemodynamics were recorded. Hypotension was defined as a decrease in systolic blood pressure to 80% of baseline or less and was treated with 6 mg ephedrine given intravenously. Bradycardia, defined as HR <60 beats per minute, was corrected using 0.6 mg of IV atropine sulfate. For the first 20 minutes intraoperatively, vital signs were taken every two minutes, then every five minutes for the next 60 minutes, and finally every 10 minutes until the procedure was over. Following surgery, the pain score was determined utilizing the visual analogue pain scale (VAS), which has a range of 0-10 (10 represents the most intense pain, and 0 represents no pain). It was first recorded every one hour for two hours, then every two hours for the next eight hours, and finally every four hours until 24 hours after the procedure. When the VAS was more than 4, inj. diclofenac 75 mg was given IV as rescue analgesia. During the postoperative phase, it was observed when the first dose of rescue analgesia was given. Any adverse event that occurred during the procedure was noted and managed appropriately.

Statistical analysis

The standard deviation (SD) and mean of collected numerical data were displayed, while counts and percentages were used to display categorical data. The Statistical Product and Service Solutions (SPSS, version 20.0.2.0; IBM SPSS Statistics for Windows, Armonk, NY) program was used to conduct statistical analysis. The sample power computed by Kraemer et al. [12] utilizing the recommended figure was used. Our research sample size was selected based on the previous research by Malhotra et al. [13]. The size of our sample was 40 patients per group with a 95% confidence interval, type II error risk of 10%, power of 0.90, effect size of 0.961, α = 0.05, and β = 0.10. The data were calculated using the mean ± SD, median, frequencies (number of cases) and percentage. The quantitative variables between research groups were compared utilizing an ANOVA test with least significant difference (LSD) post-hoc analysis for parametric data and a Kruskal-Wallis H test for non-parametric data. Chi-squared (χ2) test was used to compare categorical data, and when the anticipated frequency was 5 or fewer, the exact test was used. A p-value below 0.05 was considered statistically significant [14].

## Results

**Figure 1:**
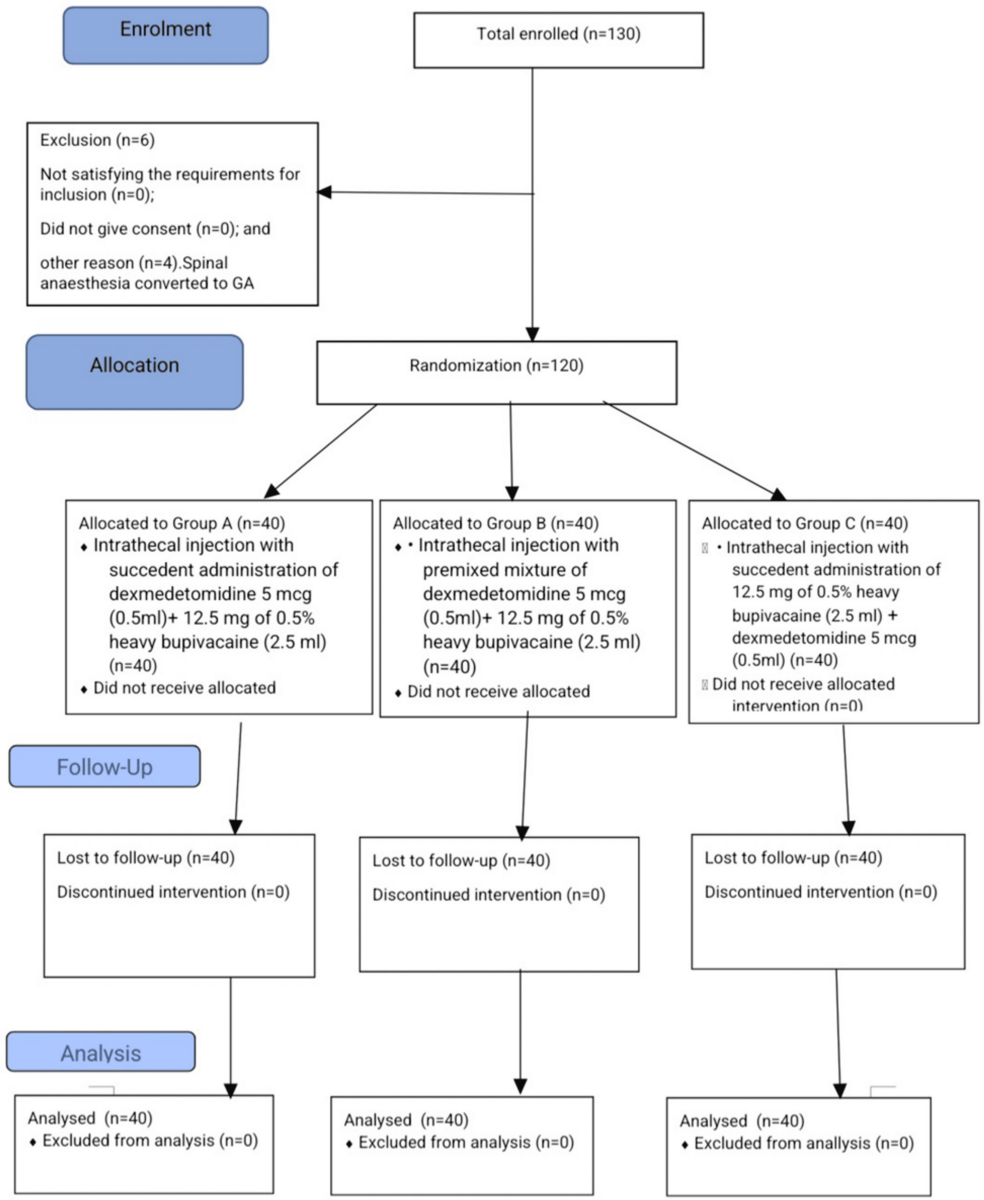
Study's CONSORT flow diagram CONSORT: CONsolidated Standards Of Reporting Trials

Table 1 presents the demographic profile of all study subjects across the three groups, which were comparable across all three groups. Figure 1 shows the study's CONsolidated Standards Of Reporting Trials (CONSORT) flow diagram.

**Table 1:** Demographic data of the study subjects in all three groups (n =120) The data were presented as mean±SD or n (%). SD=Standard deviation, ASA=American Society of Anesthesiologists. Age, Height, Weight, BMI ‑ Parametric ANOVA test. Gender, ASA ‑ Chi-square test

Variables	Group A (n = 40)	Group B (n = 40)	Group C (n = 40)
Age (years)	40.75 ± 9.34	40.75 ± 9.48	40.33 ± 9.79
Height (cm)	1.62 ± 0.05	1.62 ± 0.73	1.62 ± 0.06
Weight (kg)	63.68 ± 8.16	63.60 ± 8.92	61.38 ± 5.69
BMI (kg/m^2^)	26.0 ± 2.9	25.2 ± 2.5	26.2 ± 4.5
Gender			
Male	35 (87.5)	32 (80)	31 (77.5)
Female	5 (12.5)	8 (20)	9 (22.5)
ASA			
I	31 (77.5)	29 (72.5)	28 (70)
II	9 (22.5)	11 (27.5)	12 (30)

Table 2 displays the variations in sensory and motor block characteristics among the three groups. When three groups were compared, the sensory block started in Groups A and C earlier than in Group B. With the p-value of 0.0001, the ANOVA test illustrated a statistically significant difference between the three groups. In Groups A, B, and C, the average time to reach maximal sensory block was 10.58±19 minutes, 11.90±1.63 minutes, and 10.38±1.63 minutes, respectively. The ANOVA test represents a significant difference at a p-value of 0.001. Mean two-segment regression time from maximum level (T6) of sensory block reached 124.5±16.0 minutes in Groups A, B, and C; 114.75±15.18 minutes in Group B; and 124.25±15.67 minutes in Group C. The p-value of 0.028 indicates that the difference was statistically significant. Groups A and C experienced the onset of motor block earlier than Group B. The ANOVA test indicates a statistically significant difference with a p-value of 0.0001. Groups A, B, and C had motor block regression for an average of 317.5±32.6, 259.08±28.65, and 329.75±32.85 minutes, respectively. P-value of 0.000 was found statistically significant using the ANOVA test. Mean duration to the initial rescue analgesia requirement was 380.5±36.51 minutes for Group A, 352.35±43.17 minutes for Group B, and 352.35±43.17 minutes for Group C, which was statistically significant (p-value 0.001). LSD post-hoc analysis between Groups A and B was statistically significant, as indicated by the p-value of 0.002. Groups B and C appeared to be statistically significant based on the p-value of 0.015. A difference between Groups A and C was statistically significant, as shown by a p-value of less than 0.001.

**Table 2:** Distribution of subjects according to sensory and motor block characteristics among the study subjects of the three groups (N=40 in each group) ANOVA test applied between the three groups and t-test applied intergroups. p-value <0.05 was considered statistically significant.

Variables	Mean ± SD			p-value	p-value			t-value		
	Group A	Group B	Group C		A vs B	A vs C	B vs C	A vs B	A vs C	B vs C
Sensory onset (T10) (mins)	4.41±0.57	4.92±0.93	4.3±0.57	0.0001	0.01	0.46	0.002	2.56	0.74	3.11
Time to reach the highest level of sensory block (T6) (min)	10.58±1.29	11.90±1.63	10.38±1.63	0.001	0.01	0.60	<0.05	2.62	0.53	3.1
Motor onset (mins)	6.36±0.69	7.52±0.94	6.18±0.68	0.0001	0.001	0.31	0.001	5.45	1.02	6.32
Two segment regression of sensory level (min)	124.5±16.0	114.75±15.18	124.25±15.67	0.028	0.018	0.30	0.001	2.42	1.05	2.75
Regression of modified Bromage score to 0 (min)	317.5±32.6	259.08±28.65	329.75±32.85	0.000	0.01	0.15	<0.001	7.37	1.45	8.88
Time to first requirement of analgesia (min)	380.5±36.51	352.35±43.17	400.7±25.15	0.001	0.002	0.015	<0.001	3.15	2.49	6.13

**Figure 2:**
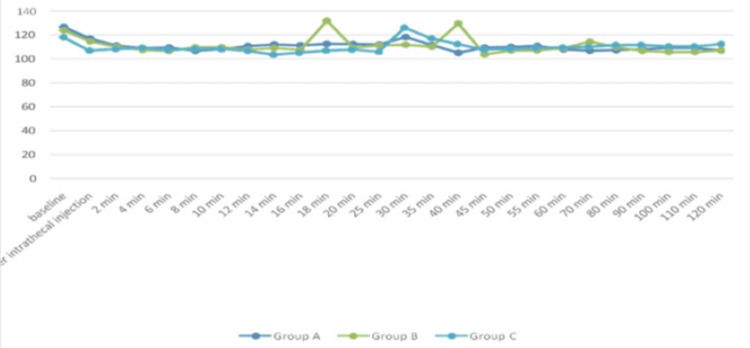
Mean heart rate (per minute) trend for each of the three groups of research participants at various times (n=40 in each group) F-test for ANOVA applied. p-value <0.05 is considered statistically significant.

The average HR for each of the three groups of study participants throughout different periods is shown in Figure 2. The mean HR at various time intervals among three groups was examined using an F-test for ANOVA, which, except for baseline and the immediate post-intrathecal injection (p-value>0.05), illustrates a statistically significant difference as p-value <0.05 at all time points.

**Figure 3:**
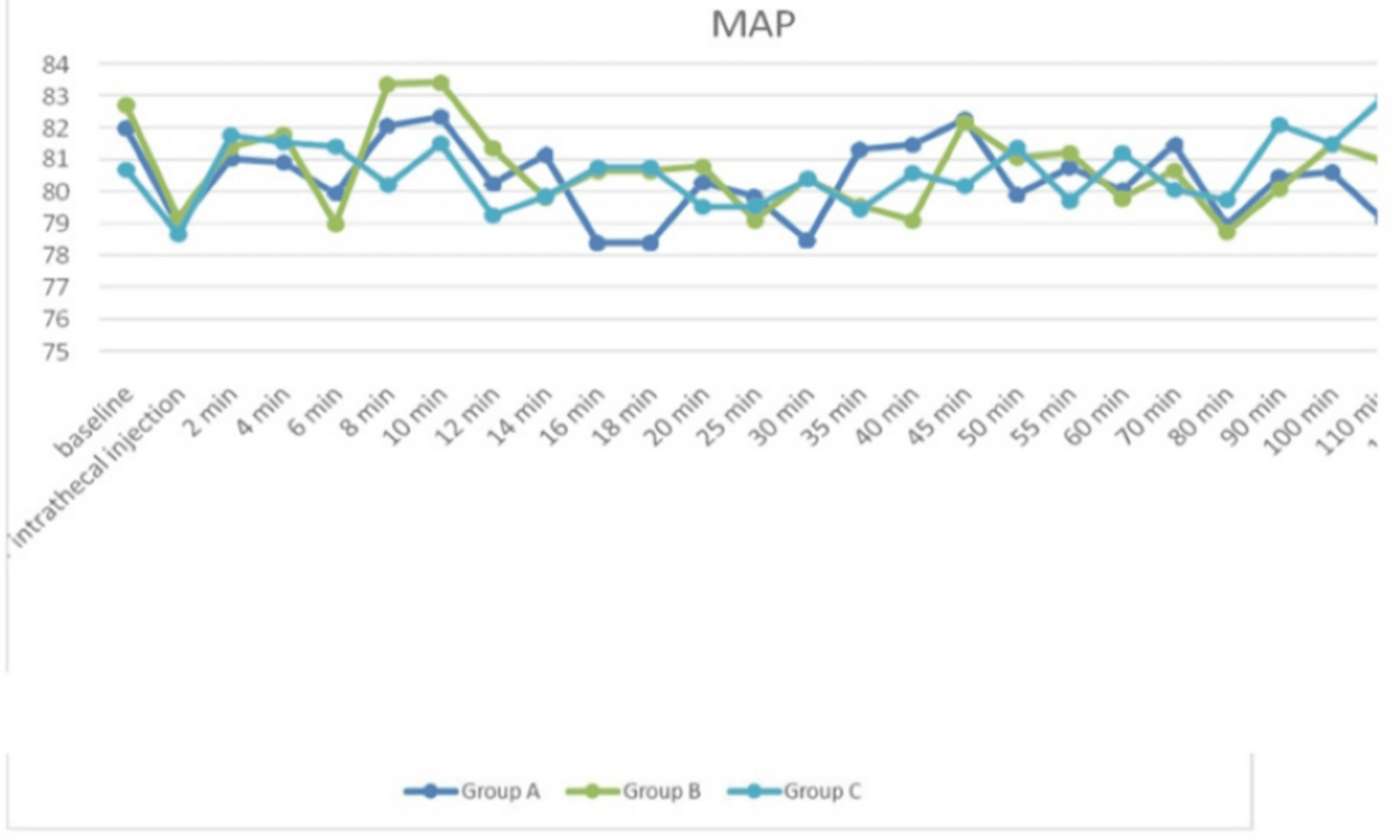
Comparison of three groups' mean arterial pressure (mmHg) trend throughout various time periods (N=40 in each group) F-test for ANOVA applied. p-value <0.05 is considered statistically significant. MAP: Mean arterial pressure

Figure 3 displays MAP for each of the three research groups at various points in time. An F-test for ANOVA has been performed to see whether there are significant differences in the mean values at different time intervals among three groups, suggesting that the differences had no significance as p-value > 0.05.

**Figure 4:**
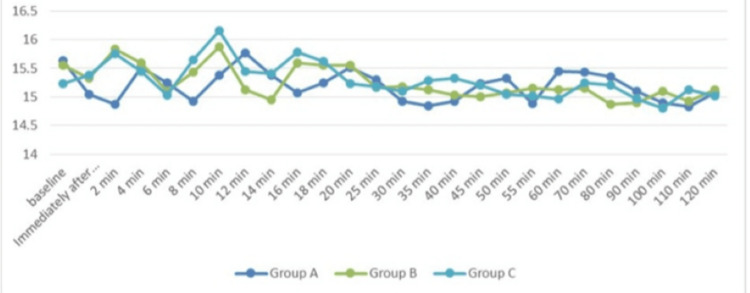
Comparison of the respiratory rate (per minute) trend of the three groups (N=40 in each group) at different time periods F-test for ANOVA applied. P-value <0.05 is considered statistically significant.

Figure 4 illustrates the respiratory rates over different periods of time across the three study groups, resulting in a statistically insignificant difference as p-value > 0.05 at all time intervals.

**Figure 5:**
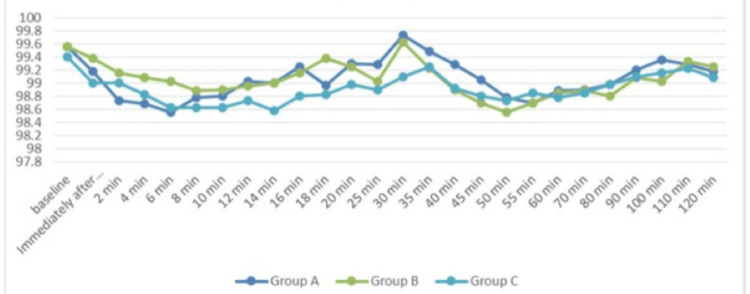
Comparison of the mean SpO2 (%) trend over different periods among the study subjects (N=40 in each group) F-test for ANOVA applied. p-value <0.05 is considered statistically significant.

Figure 5 illustrates oxygen saturation (SpO_2_) at different time periods among the three study groups, and the difference was not significant as p-value >0.05 at all time intervals.

**Figure 6:**
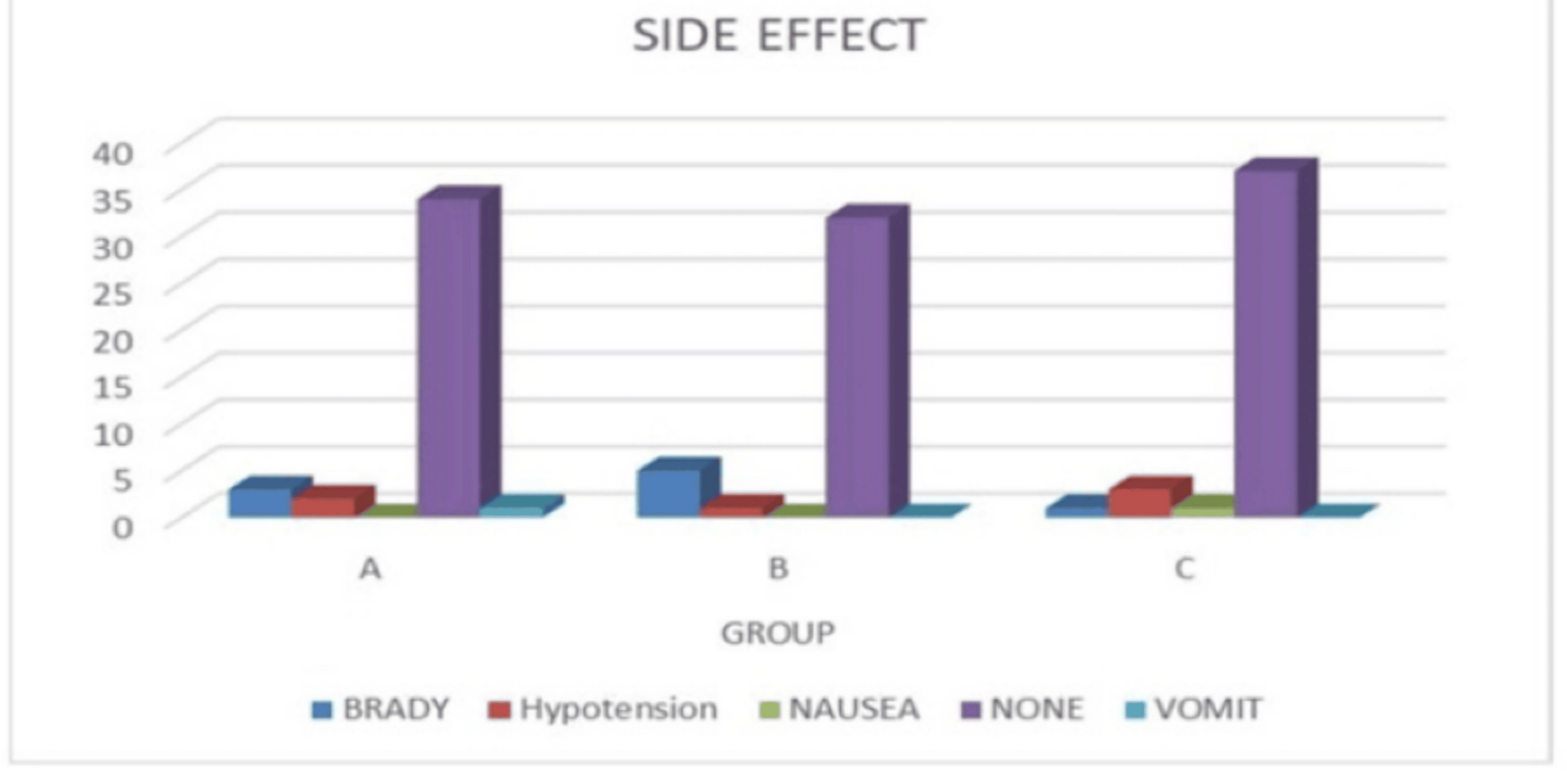
Side effects among the study subjects from three groups (N=40 in each group) Chi-square test applied. p-value <0.05 is considered statistically significant. Chi-square value: 8.03, p-value: 0.43, non-significant.

Figure 6 illustrates the adverse effects experienced by the patients in three groups. Three patients in Group A experienced bradycardia, two were diagnosed with hypotension, and one had vomiting. Five patients of Group B had bradycardia, whereas one had hypotension, one in Group C suffered bradycardia, one had nausea, and one had hypotension. However, a non-significant difference was revealed by the comparison (p-value=0.43).

## Discussion

Spinal anaesthesia is the most often used method for delivering anaesthesia during infraumbilical surgeries. Since spinal anaesthesia utilizing only local anesthetics has a short-term analgesic effect, post-operative pain relief is a concern. Early analgesic treatment is therefore necessary during the post-operative phase [6]. Intrathecal local anaesthetics are commonly used with the right adjuvants to enhance blockade quality, prolong analgesia, and reduce the dosage of local anaesthetics. This lowers the risk of hypotension, nausea, vomiting, and late and severe bradycardia, all of which are adverse consequences of high-dose local anaesthetics [15].

A highly selective agonist of alpha-2 adrenoreceptors, dexmedetomidine acts like a central sympatholytic by blocking presynaptic terminals from releasing noradrenaline. Its analgesic, sympatholytic, and sedative properties have demonstrated its utility in numerous anaesthetic and critical care settings. Additionally, its potential as a local anaesthetic adjuvant in spinal as well as regional anaesthesia is being explored. The impact of mixing dexmedetomidine with intrathecal local anaesthetics has been evaluated in several studies [16,17]. Dexmedetomidine has been found to prolong analgesia with limited adverse effects. According to theory, it attaches to presynaptic C fibres, as well as hyperpolarises postsynaptic dorsal horn neurons, which block the release of neurotransmitters, though the precise mechanism underlying this action is unclear. It is believed that this action works by hyperpolarising postsynaptic dorsal horn neurons, as well as attaching to presynaptic C fibres, which inhibit the release of neurotransmitters, though the precise process is unknown. Additionally, it has been observed to decrease the frequency of shivering during the recovery period and the requirement for post-anaesthetic analgesics. Similarly, optimal intrathecal dosage of dexmedetomidine has been studied [17,18].

According to a meta-analysis by Liu et al. [19], intrathecal delivery of 5 mcg of dexmedetomidine resulted in the onset of prolonged motor blockade and sensory-motor blockade. It increased the time before the first request for rescue analgesia. A randomised clinical trial including 90 patients having lower limb elective procedures, by Faiz et al. [20], found similar outcomes. Therefore, we utilised 5 mcg of dexmedetomidine per dosage, reconstituted with 0.9% normal saline that was free of preservatives as a spinal anaesthesia adjuvant in our research.

Usage of adjuvants in premixed combination with local anaesthetics in a single syringe for intrathecal injection may change the density of the resulting solution. Research conducted by Imbelloni et al. [21] demonstrates that the incorporation of adjuvants into local anaesthetics reduces the density of the resulting solution. A 0.0006 g/mL change in density is a significant alteration that affects the dispersion of LA in CSF. We hypothesized that using distinct syringes to provide adjuvants and bupivacaine in sequence would produce superior block characteristics than the premixed solution delivery in a single syringe.

In our study, the succedent groups had earlier sensory and motor block onset than the premixed group, and this difference was statistically significant. No significant differences had been observed among sequential Groups A and C in intergroup comparison. The succedent groups had reached the highest sensory block level earlier than the premixed group, and with a p-value of 0.001, the difference was statistically significant. Although no significant difference was seen among the succedent groups. We also discovered that there was a longer sensory and motor block in the succedent groups, which was statistically significant than the premixed group. Results from research by Chaudhary et al. [9] that used different dosages of dexmedetomidine as an adjuvant were consistent with our findings. However, no significant difference among the succedent groups, which differs from the results of research conducted by Malhotra et al. [13] on lower limb procedures involving fentanyl as an adjuvant with bupivacaine. Similar research has been conducted by Singh et al. [22] on patients scheduled for lower abdominal surgery who received a similar dosage of heavy bupivacaine and dexmedetomidine. They discovered that the sequential group had motor blockage earlier than the premixed group. Similar findings were seen in our investigation as well. We found that the succedent groups did not differ significantly from one another. Several studies had results similar to our results [10,21].

It is easier to distribute dexmedetomidine and heavy bupivacaine when they are administered in different syringes. It thus establishes an additional bond with receptors, resulting in a denser and more sustained block, compared to a less effective block accomplished by a diluted combination of hyperbaric bupivacaine and dexmedetomidine. Additionally, Gray et al. [23] discovered that hypobaric morphine produces analgesia for a longer period of time than hyperbaric morphine.

In our research, we discovered that the average HR changes in all three groups were not significant at baseline or just after intrathecal injection, but they were significant at all subsequent time points. The magnitude of decrease in HR was greater in subsequent groups than in the premixed group. The findings aligned with the research done by Chekole et al. [24] and Singla et al. [25]. MAP had no significant differences across each group. After the intrathecal injection, there was a drop in MAP; however, the premixed group experienced a much smaller drop in MAP at 12 minutes than the succedent groups. This outcome aligns with what Keera et al. discovered [26] in their research. Additionally, changes in SpO_2_ and RR were not significant across all groups. Sachan et al. additionally found that the premixed group's time for first rescue analgesia was considerably shorter than that of the succedent groups [10].

In our study, out of the following three groups, the time to the first requirement of analgesia (min) was the highest in Group C as compared to Group A and Group B, and it was statistically significant.

In all three groups, there were very few adverse effects that were statistically insignificant. Among the adverse effects, bradycardia was more common. When dexmedetomidine is given intrathecally, it inhibits shivering by activating alpha-2 adrenergic receptors, which decreases sympathetic outflow and lowers the shivering threshold; therefore, shivering is usually absent. Our research observed neither severe hypotension nor severe bradycardia. Similar outcomes have been observed in research conducted by Gupta et. al [4] and Mahendru et. al [27], which showed dexmedetomidine as an attractive adjuvant to spinal ropivacaine or bupivacaine in long-duration surgical procedures due to its profound intrathecal anaesthetic and analgesic properties combined with minimal side effects.

During our research, we saw several limitations, such as the inability to measure the specific gravity of medications and CSF, the inability to measure the density of premixed solutions of drugs to predict their spread into CSF, and the failure to keep the study drugs' temperature. Additional research is necessary because this study has been conducted for a short period of time and in a small sample size.

## Conclusions

Succedent groups had an earlier onset of motor and sensory block than the premixed group. Sequential groups experienced a longer length of sensory and motor block than the premixed group. SpO_2_, RR, and MAP had no significant difference in any of the three groups. Still, hemodynamically, the succedent groups showed substantial reductions in HR after intrathecal injection compared to the premixed group. Compared to the premixed group, the duration of initial rescue analgesia was longer in the succedent groups. Consequently, we ascertain that the initiation and persistence of sensory and motor block attributes in spinal anaesthesia are markedly enhanced by the subsequent groups as compared to the premixed groups. As compared to the premixed group, a lower incidence of hypotension was seen in the sequential groups, and there was no significant difference among the sequential groups. None of the three groups experienced any statistically significant adverse side effects.
